# Causal Effects of Immune Cell Populations on Cognitive Performance: A Mendelian Randomization Study

**DOI:** 10.1002/brb3.70861

**Published:** 2025-09-10

**Authors:** Jingfeng Fu, Minmin Yang, Qingteng Zheng

**Affiliations:** ^1^ School of Pharmacy and Medical Technology Putian University Putian China; ^2^ Key Laboratory of Pharmaceutical Analysis and Laboratory Medicine Putian University Putian China

**Keywords:** cognitive performance, genome‐wide association study, immune cells, Mendelian randomization

## Abstract

**Background:**

Recent research has started to uncover an important connection between immune system activity and cognitive abilities. Although correlative associations have been documented, the causal mechanisms connecting specific immune cell subpopulations to cognitive capabilities remain insufficiently characterized. Our research aimed to determine directional relationships between distinct immune cell subtypes and cognitive function, potentially identifying targets for immunomodulatory interventions.

**Methods:**

We performed a two‐sample Mendelian randomization (MR) analysis using genome‐wide association study data from 3757 Sardinian individuals, paired with detailed immunophenotyping. We also incorporated cognitive performance summary statistics from the cohort described by Lee et al. (*n* = 257,841). Our analytical strategy utilized various MR techniques, with inverse variance weighted analysis serving as the primary method. To confirm result reliability, we conducted sensitivity analyses, including weighted median estimation, mode‐based approaches, MR‐Egger regression for evaluating pleiotropic effects, MR‐PRESSO for outlier identification, and Cochran's *Q*‐statistic to examine heterogeneity. Additionally, to explore possible reverse causation mechanisms, we conducted bidirectional MR analyses.

**Results:**

Post false discovery rate (FDR) correction (*P*_FDR_ < 0.05), we identified two immune cell phenotypes significantly linked to cognitive performance. *IgD^−^ CD27^−^ B cells %lymphocyte* showed a positive correlation with cognitive outcomes (*β* = 0.04, 95% confidence interval [CI]: 0.02–0.06, *P*_FDR_ = 3.02 × 10^^−2^), whereas *unswitched memory B cells %lymphocyte* demonstrated negative association (*β* = −0.06, 95% CI: −0.09 to −0.03, *P*_FDR_ = 3.02 × 10^^−2^). When applying stricter statistical thresholds (*p* < 0.005), five distinct immune subpopulations demonstrated significant relationships: among B‐lymphocytes (*IgD* ^−^ *CD27* ^−^ *B cells*, *CD27^+^ memory B cells*, and *CD38^+^ transitional B cells*), T‐lymphocytes (*CCR7^+^ naive CD4^+^ T cells*), and mononuclear phagocytes (*HLA‐DR^+^ CD14^−^ CD16^−^ cells*). These findings reveal distinct immunophenotypic signatures potentially influencing cognitive function through various cellular pathways. Importantly, bidirectional MR analyses revealed no significant causal effects of cognitive performance on these immunophenotypic signatures, strengthening the directionality of our primary findings.

**Conclusion:**

These findings suggest that seven distinct immune cell phenotypes may play a causal role in cognitive functioning. The absence of reverse causality further supports that these immunophenotypes likely influence cognitive outcomes rather than being consequences of cognitive function. The identified causal associations indicate potential immune pathways that could be relevant for modulating cognitive function. These immune signatures may serve as key regulators in cognitive performance‐associated inflammatory pathways.

## Graphical abstract

This Mendelian randomization study analyzed immune cell populations and identified potential causal links between immune profiles and cognitive performance, offering insights into immune mechanisms underlying cognition.

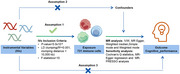

**Abbreviations**
CI: confidence interval
FDR: false discovery rate
GWAS: genome‐wide association study
IV: instrumental variable
IVW: inverse variance weighted
LD: linkage disequilibrium
MR: Mendelian randomization
SNPs: single‐nucleotide polymorphisms
*β*
: beta coefficient

## 1 Introduction

Human cognitive functionality integrates multiple processes for knowledge application, including sensory processing, attention, memory, reasoning, and linguistic capabilities (Chen et al. 2022). Neurocognitive disorders and dementia represent major contributors to global health burden, particularly amid demographic aging (Livingston et al. 2020). Various factors influence cognitive performance, including environmental conditions, psychological states, aging processes, neurological conditions, hormonal variations, and immune system alterations (Elias et al. 1995; Martin et al. 2019; Williams et al. 2019). With dementia affecting 47 million individuals globally in 2015 and projections suggesting a tripling by 2050, developing novel interventions for cognitive decline prevention has become imperative. Notably, immunological dysregulation demonstrates significant correlation with cognitive deterioration. Evidence increasingly indicates elevated cognitive dysfunction prevalence in patients with immune‐mediated inflammatory conditions (Gwinnutt et al. 2023).

Cognitive decline manifests as a continuous, progressive, and irreversible process. Key accelerating factors include chronic inflammation and cellular senescence, both substantially regulated by immune cells (Murdock et al. 2022; Trollor et al. 2012). The significance of immune cell involvement in cognitive dysfunction has received growing attention (Derecki et al. 2011). In healthy elderly populations, cognitive performance correlates with peripheral immune markers—a cross‐sectional investigation demonstrated that individuals with diminished cognitive abilities exhibited elevated levels of interleukin‐1*β*, interleukin‐8, interleukin‐13, and tumor necrosis factor (TNF) compared to their cognitively superior counterparts (Serre‐Miranda et al. 2020). During normal brain aging and cognitive deterioration, cerebrospinal fluid immune dysregulation becomes apparent, characterized by C–X–C motif chemokine receptor 6 (CXCR6) upregulation on clonal CD8 effector memory T cells in cognitively impaired individuals (Piehl et al. 2022). Additionally, CD4^+^ T cells contribute to neurodegenerative processes in Lewy body dementia (Gate et al. 2021). Patients with mild cognitive impairment display higher proportions of regulatory T cells relative to those with Alzheimer's disease‐related dementia (Fu et al. 2021). These collective findings suggest substantial influence of circulating immune components on cognitive function pathogenesis, though the directional nature of these relationships requires further clarification.

Among the key factors accelerating cognitive deterioration are chronic inflammation and cellular senescence, both significantly modulated by immune cells (Murdock et al. 2022; Trollor et al. 2012). The pivotal role of immune cells in cognitive dysfunction disorders has gained increasing recognition (Derecki et al. 2011). For example, cognitive performance in healthy elderly individuals is correlated with peripheral immune markers. A cross‐sectional study demonstrated that participants exhibiting poorer cognitive performance had elevated concentrations of interleukin‐8, interleukin‐1*β*, interleukin‐13, and TNF compared to those with superior cognitive function (Serre‐Miranda et al. 2020). Cerebrospinal fluid immune dysregulation has been observed during normal brain aging and cognitive impairment processes, with upregulation of CXCR6 on clonal CD8 effector memory T cells in subjects with cognitive impairment (Piehl et al. 2022). Furthermore, CD4^+^ T cells contribute to neurodegeneration in Lewy body dementia (Gate et al. 2021). Notably, patients with mild cognitive impairment demonstrate higher regulatory T‐cell proportions compared to patients with Alzheimer's disease‐related dementia (Fu et al. 2021). The accumulating scientific literature suggests that circulating immune cells may exercise substantial influence over cognitive function pathogenesis. Nevertheless, the exact nature of the relationship between systemic immune cell populations and cognitive function remains inadequately characterized, thereby necessitating further investigation.

Mendelian randomization (MR) methodology offers a sophisticated approach to addressing the limitations of traditional observational research and has emerged as a valuable tool for investigating causal relationships between specific exposures and outcomes through the use of genetic instrumental variables (IVs) (Lawlor 2016; Sanderson et al. 2022). MR leverages the random allocation of single‐nucleotide polymorphisms (SNPs) during embryonic development, thereby creating natural experiments that reduce confounding influences (Lawlor et al. 2008; Sekula et al. 2016; Zheng et al. 2017). This framework approximates randomized controlled trial conditions while utilizing genomic data. Although previous MR studies have established causal links between immune system components and overt neurodegenerative diseases such as Alzheimer's (Chang et al. 2024; Xue et al. 2024), a critical knowledge gap remains regarding the immune system's role in modulating general cognitive function across the non‐pathological spectrum. Our research diverges from prior work by conceptualizing cognitive performance not as a binary disease state, but as a continuous trait. This approach enables the identification of immune pathways that influence cognitive variability before the onset of clinical dementia, thereby shifting the scientific focus from disease treatment to strategies for primary prevention and the maintenance of lifelong cognitive health. Therefore, this study leverages the MR framework to systematically investigate the causal effects of specific immune cell phenotypes on cognitive performance, a crucial yet underexplored area.

Our study employs a two‐sample MR approach to systematically evaluate the causal relationships between circulating immune cell phenotypes and risk factors for cognitive impairment. Identifying the causal contributions of peripheral immune components along with their inflammatory mechanisms may reveal promising therapeutic targets and diagnostic biomarkers for early detection and intervention monitoring. Ultimately, these insights could enhance our understanding of cognitive improvement strategies through targeted immunomodulation.

## 2 Materials and Methods

### 2.1 Study Design

**FIGURE 1:**
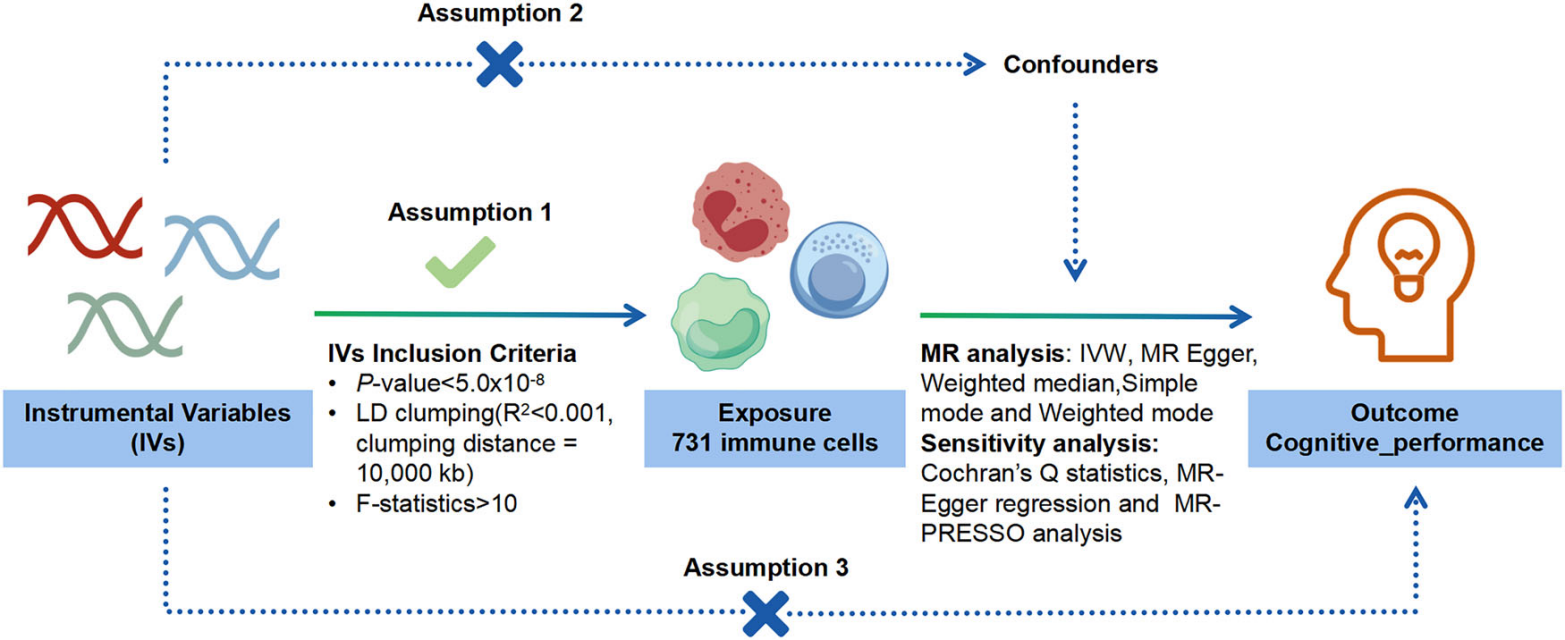
Schematic Illustration of the MR Analysis. IVW, inverse variance weighted; MR, Mendelian randomization.

Our research employed a comprehensive two‐sample MR approach to investigate potential causal relationships between 731 immune cell profiles and cognitive function. To ensure methodological validity, we followed three fundamental MR assumptions: (1) The IVs showed strong correlations with exposure variables, meeting the relevance criterion. (2) The selected genetic instruments functioned independently of confounding factors that might influence both exposure and outcome measurements, satisfying the independence criterion. And (3) the genetic instruments affected the outcome exclusively through their effect on exposure, without involving alternative causal pathways, thus fulfilling the exclusion restriction (Boef et al. 2015; de Leeuw et al. 2022). The analytical framework is depicted in Figure 1. This investigation was performed in complete accordance with the Strengthening the Reporting of Observational Studies in Epidemiology using Mendelian Randomization (STROBE‐MR) guidelines, with detailed compliance documentation provided in Table S1 (Skrivankova et al. 2021).

### 2.2 Genome‐Wide Association Studies (GWAS) Data Sources for Immune Cell Phenotypes

For our investigation, we leveraged the most extensive immunophenotyping GWAS data available, featuring 731 distinct immune signatures characterized in 3757 Sardinian individuals of European ancestry (Sidore et al. 2015). This comprehensive profiling encompassed multiple cellular metrics, including: 118 absolute cell (AC) quantifications, 389 surface antigen expression measurements via median fluorescence intensities (MFIs), 32 parameters derived from forward scatter (FSC) and side scatter (SSC) measurements reflecting cellular morphology, and 192 relative cell (RC) population assessments. Approximately 22 million SNPs were examined through direct high‐density array genotyping or imputation using Sardinia‐specific reference panels. Age and sex were incorporated as covariates throughout all analyses to control for potential demographic confounding. The complete dataset containing summary statistics for all 731 immunological phenotypes is accessible through the GWAS Catalog (identifiers GCST0001391–GCST0002121) (Orrù et al. 2020).

### 2.3 GWAS Data Sources for Cognitive Performance

Our cognitive performance analyses incorporated extensive genetic association data compiled by the Social Science Genetic Association Consortium (SSGAC), encompassing 257,841 participants of European ancestry (Lee et al. 2018). This comprehensive repository integrates prior Cognitive Genomics Consortium (COGENT) findings (35,298 individuals) (Trampush et al. 2017) with UK Biobank (UKB) cognitive assessment data (222,543 subjects). The merged statistical repository demonstrated substantial explanatory capacity, explaining 7%–10% of observed variation in cognitive measurement outcomes.

### 2.4 Selection of IVs

In our IV selection process, we implemented a genome‐wide significance threshold of *p* < 5 × 10^^−8^ for each immunophenotype, aligning with methodological standards in previous MR studies of immune parameters (Xu et al. 2024). To ensure independent predictive capacity, we performed linkage disequilibrium (LD) clumping using the 1000 Genomes Project reference panel with stringent parameters (*r* ^2^ < 0.001 within 10 Mb genomic windows) (Purcell et al. 2007). We systematically assessed IV strength by calculating both the proportion of phenotypic variation explained (PVE) and corresponding *F*‐statistic to address potential weak instrument bias (Palmer et al. 2012). Instruments yielding *F*‐statistics below 10 were classified as weak and excluded from subsequent analyses. *F*‐Statistics were computed using the formula *F* = (*β*/SE)^2^, where *β* represents the effect estimate magnitude and SE denotes its standard error. The proportion of phenotypic variation (*R* ^2^) was calculated using *R* ^2^ = 2 × EAF × (1 − EAF) × *β* ^2^, where EAF represents effect allele frequency and *β* indicates the estimated SNP effect on the phenotypic trait (S. Burgess et al. 2011; Lv et al. 2023; Pierce et al. 2011).

### 2.5 MR Analysis

The comprehensive statistical procedures were performed using the R computing environment (version 4.4.1). We assessed potential causal links between 731 immune cell traits and cognitive function through multiple MR techniques implemented via the “MendelianRandomization” package (version 0.6.6) (Yavorska and Burgess 2017). The analytical strategy incorporated several complementary methods, including inverse variance weighted (IVW) (S. Burgess et al. 2015), simple mode (Boehm and Zhou 2022), weighted mode (Hartwig et al. 2017), weighted median (Bowden et al. 2016), and MR‐Egger regression (Bowden et al. 2015), with IVW functioning as the primary analytical framework. For IVW analyses, the choice between fixed‐effect and random‐effect models was determined by heterogeneity evaluation results: Fixed‐effect models were applied when significant heterogeneity was not present, whereas random‐effect models were utilized when heterogeneity was observed (Bowden et al. 2017).

### 2.6 Statistical Power Analysis

Post hoc statistical power for our significant findings was determined using the mRnd online calculator (http://cnsgenomics.com/shiny/mRnd/). As our outcome was a continuous variable, the power for each exposure‐outcome pair was estimated on the basis of (1) the proportion of variance in the exposure explained by the IVs (*R* ^2^); (2) the sample size of the cognitive performance GWAS; (3) the causal effect size estimated in our MR analysis; and (4) a Type I error rate (*α*) set at 0.05. The *R* ^2^ for each instrumental SNP was computed with the formula: *R* ^2^ = 2 × EAF × (1 − EAF) × *β* ^2^, where EAF represents the effect allele frequency and *β* is the effect of the SNP on the exposure. The cumulative *R* ^2^ for multi‐SNP instruments was calculated by summing the individual *R* ^2^ values.

### 2.7 Sensitivity Analyses

To verify result reliability, we conducted extensive sensitivity evaluations, including Cochran's *Q* test (S. Burgess et al. 2013) for measuring heterogeneity across IVs, MR‐Egger intercept assessment (Bowden et al. 2015) for detecting horizontal pleiotropy (with *p* > 0.05 suggesting no significant directional pleiotropy), and MR‐PRESSO approach (Verbanck et al. 2018) for identifying and removing potential pleiotropic outliers. Several immune cell traits were unavoidably excluded from sensitivity analyses due to insufficient genetic instrumentation (fewer than three genome‐wide significant SNPs) in the immunophenotyping GWAS dataset (Harrison et al. 2020).

### 2.8 Reverse MR Analyses

To investigate potential bidirectional relationships, we conducted reverse MR, evaluating whether cognitive performance exerts causal effects on the previously identified significant immune cell populations. For this reciprocal analysis, we selected SNPs showing association with cognitive performance (*p* < 5.0 × 10^^−8^) as IVs.

### 2.9 Ethics Approval and Consent to Participate

Our study utilized previously published, publicly available GWAS summary statistics. Formal ethical approval was not required, as our analysis employed exclusively summary‐level data without access to individual‐level information.

## 3 Results

### 3.1 Selection of IVs

Our investigation initially identified 2326 SNPs linked to immune cell traits at genome‐wide significance (*p* < 5 × 10^^−8^) after completing LD clumping and harmonization steps. To maintain IV validity, we implemented a rigorous filtering protocol. We excluded palindromic and ambiguous alleles to avoid strand ambiguity complications. SNPs lacking sufficient strength (*F*‐statistics <10) and those exhibiting strong correlations with cognitive measures were removed to reduce potential bias. After pleiotropy evaluation using MR‐PRESSO, we eliminated SNPs showing pleiotropic effects. This thorough quality control process yielded 2318 valid IVs for our final MR analysis examining the connection between immune cell profiles and cognitive function, with full details available in Table S2.

### 3.2 Causal Influences of Immune Cell Profiles on Cognitive Function

Using two‐sample MR with IVW analysis as our main statistical approach, we investigated potential causal links between 731 different immune cell profiles and cognitive function. Following adjustment for multiple testing using false discovery rate correction (*P*_FDR_ < 0.05), we identified two specific immune cell subpopulations showing statistically significant relationships with cognitive performance measures in Table S3. Post hoc power analysis confirmed that our study was well powered to detect these associations, with statistical power of 99.4% and 100%, respectively (see Table S4 for details).

**FIGURE 2:**
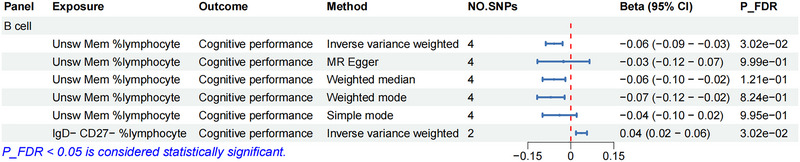
Forest plot illustrating MR analysis results depicting the relationship between immune cell traits and cognitive function (*P*_FDR_ < 0.05). CI, confidence interval; MR, Mendelian randomization; SNPs, single‐nucleotide polymorphisms.

**FIGURE 3:**
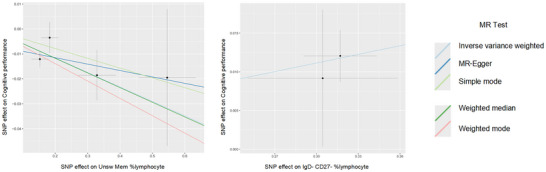
Scatter plots illustrating the MR effect of immune cell phenotypes on cognitive performance (*P*_FDR_ < 0.05). *X*‐axis shows genetic associations with immune phenotypes; *Y*‐axis shows associations with cognitive performance. Regression lines represent five MR analytical methods. Each black dot indicates an individual SNP instrumental variable, demonstrating causal effect consistency across genetic variants. MR, Mendelian randomization; SNPs, single‐nucleotide polymorphisms.

Our results showed that B cells with the *IgD^−^ CD27^−^ phenotype %lymphocyte* displayed positive correlation with cognitive outcomes (*β* = 0.04, 95% CI: 0.02–0.06, *P*_FDR_ = 3.02 × 10^^−2^), suggesting possible neuroprotective effects. In contrast, *unswitched memory B cells %lymphocyte* showed a negative association with cognitive performance (*β* = −0.06, 95% CI: −0.09 to −0.03, *P*_FDR_ = 3.02 × 10^^−2^), indicating a potentially detrimental impact on cognitive function (Figure 2). The causal relationships between these immune cell profiles and cognitive performance are depicted in the scatter plots in Figure 3, demonstrating both the direction and strength of these associations.

**FIGURE 4:**
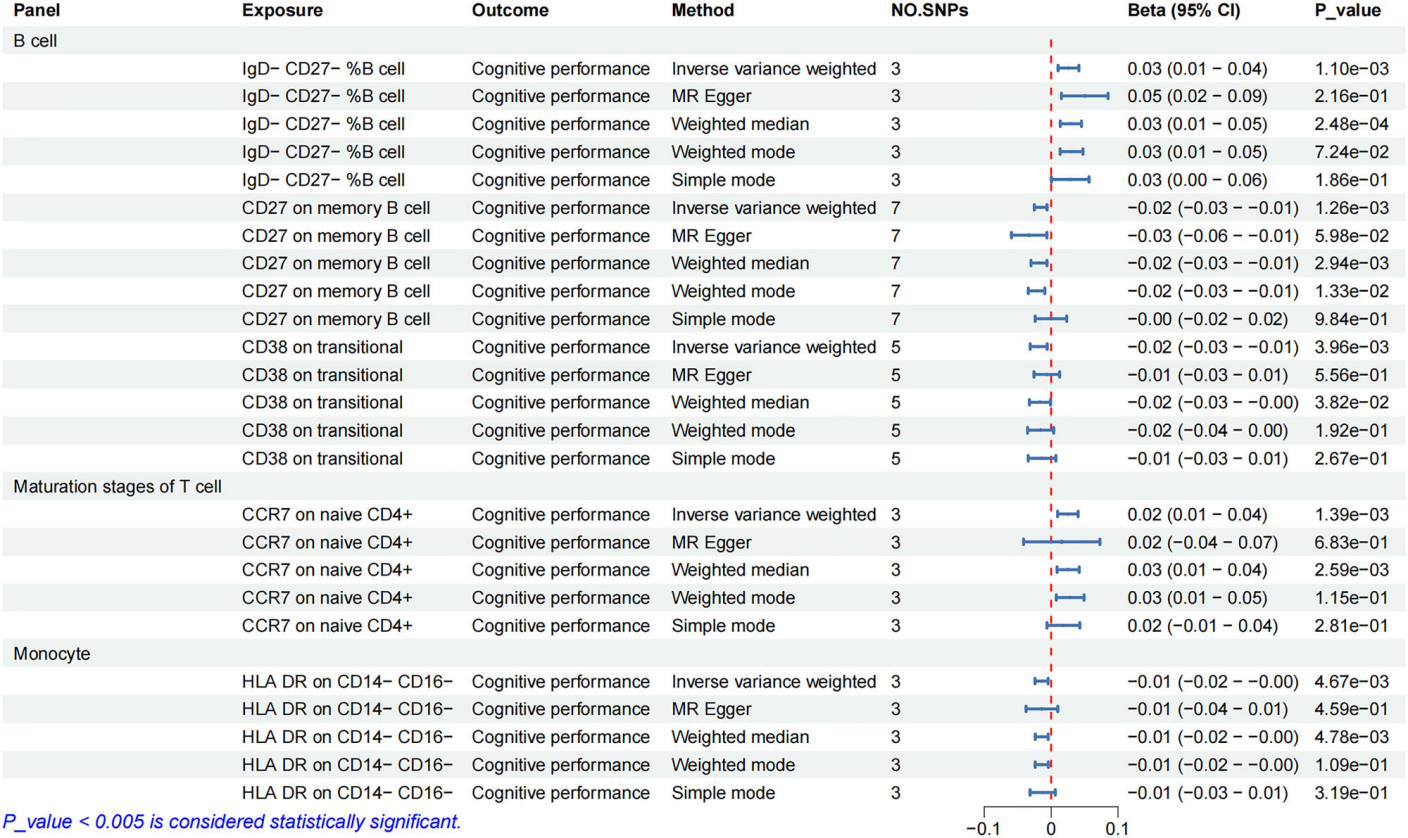
Forest plot illustrating MR analysis results depicting the relationship between immune cell traits and cognitive function (*p* < 0.005). CI, confidence interval; MR, Mendelian randomization; SNPs, single nucleotide polymorphisms.

**FIGURE 5:**
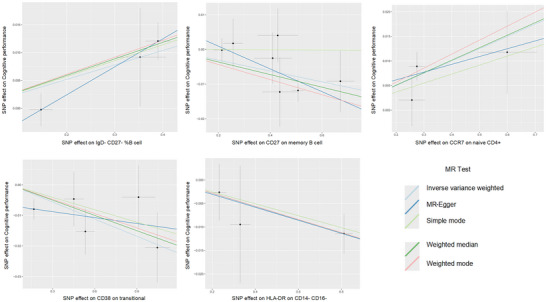
Scatter plots illustrating the MR effect of immune cell phenotypes on cognitive performance (*p* < 0.005). MR, Mendelian randomization; SNPs, single‐nucleotide polymorphisms.

When implementing a more stringent significance threshold (*p* < 0.005), five immune cell subtypes met the criteria (Table S3). The statistical power for these five findings was also robust, ranging from 85.0% to 98.2%, further strengthening confidence in these results (Table S4). Within the B cell compartment, *IgD^−^ CD27^−^ B cells* (as a percentage of B cells) demonstrated potential cognitive protective effects (*β* = 0.03, 95% CI: 0.01 to 0.04, *p* = 1.10 × 10^−3^), whereas *CD27 expression on memory B cells* (*β* = −0.02, 95% CI: −0.03 to −0.01, *p* = 1.26 × 10^−3^) and *CD38 expression on transitional B cells* (*β* = −0.02, 95% CI: −0.03 to −0.01, *p* = 3.96 × 10^−3^) were associated with potential cognitive decline. Within T cell maturation stages, *CCR7 expression on naive CD4^+^ T cells* (*β* = 0.02, 95% CI: 0.01–0.04, *p* = 1.39 × 10^−3^) exhibited protective properties. Among monocytes, *HLA‐DR expression on CD14* ^−^ *CD16* ^−^ *cells* (*β* = −0.01, 95% CI: −0.02 to −0.00, *p* = 4.67 × 10^−3^) demonstrated inhibitory effects (Figure 4). The causal relationships between these immunophenotypes and cognitive performance are visualized in the scatter plots presented in Figure 5.

### 3.3 Sensitivity Analyses

The genetic instruments employed demonstrated robust validity with *F*‐statistics exceeding the recommended threshold of 10, mitigating potential weak instrument concerns (Table S2). Following outlier removal via MR‐PRESSO (Table S5), Cochran's *Q* analysis confirmed instrumental homogeneity across remaining variants (Table S6). For analyses involving more than three SNPs, horizontal pleiotropy assessment through MR‐Egger intercept tests (Table S7) and MR‐PRESSO global evaluations (Table 1) revealed no significant directional bias. Additional confirmation of result stability was achieved through sequential variant exclusion analyses and symmetry evaluation via funnel plot visualization (Figures S1–S13), collectively strengthening the reliability of identified causal relationships between immune cell signatures and cognitive outcomes.

**TABLE 1:** Sensitivity analysis outcomes from the Mendelian randomization study evaluating cognitive performance.

Exposure	Heterogeneity analysis						Pleiotropy analysis			
	Inverse variance weighted			MR‐Egger			MR‐Egger			MR‐PRESSO global test
	*Q*	*Q*_df	*Q*_*p*val	*Q*	*Q*_df	*Q*_*p*val	Egger_intercept	se	*p*val	*p*val
Unsw Mem %lymphocyte	2.48	3.00	0.48	1.94	2.00	0.38	−0.01	0.01	0.54	0.49
IgD^−^ CD27^−^ %lymphocyte	0.07	1.00	0.80	NA	NA	NA	NA	NA	NA	NA
IgD^−^ CD27^−^ %B cell	2.28	2.00	0.32	0.01	1.00	0.91	−0.01	0.01	0.37	NA
CD27 on memory B cell	5.46	6.00	0.49	3.61	5.00	0.61	0.01	0.01	0.23	0.40
CCR7 on naive CD4^+^	1.32	2.00	0.52	1.19	1.00	0.27	0.00	0.01	0.80	NA
CD38 on transitional	4.44	4.00	0.35	1.85	3.00	0.60	−0.01	0.00	0.21	0.43
HLA DR on CD14^−^ CD16^−^	0.16	2.00	0.92	0.16	1.00	0.69	0.00	0.01	0.98	NA

Abbreviations: MR, Mendelian randomization; SNPs, single‐nucleotide polymorphisms.

### 3.4 Reverse MR Analyses

To rigorously assess potential reverse causation, we implemented bidirectional MR analyses employing SNPs associated with cognitive performance (*p* < 5.0 × 10^^−8^) as IVs. These comprehensive reverse analyses revealed no statistically significant bidirectional causal effects flowing from cognitive performance to the immunophenotypic signatures (Table S8). This absence of reverse causality reinforces the directionality of our primary observations, lending support to the interpretation that the identified immunophenotypes exert causal influence on cognitive performance rather than emerging as consequences of cognitive function or reflecting confounding factors. These findings collectively strengthen the robustness of our causal inference framework and further validate the potential biological relevance of the identified immunophenotypic markers in the modulation of cognitive outcomes.

## 4 Discussion

This investigation constitutes the first MR study examining potential causal relationships between immune cell phenotypic signatures and cognitive performance. Through our comprehensive analysis of 731 distinct immune cell profiles, we identified two that exhibited statistically significant associations with cognitive function after adjustment for FDR. Notably, *IgD^−^ CD27^−^ %lymphocyte* demonstrated positive correlations with superior cognitive outcomes, suggesting these cells may confer neuroprotective effects. Conversely, *Unsw Mem %lymphocyte* showed an inverse relationship with cognitive assessment scores, potentially indicating deleterious effects on neural function and cognitive processes.

When employing a more stringent significance threshold (*p* < 0.005), five immune cell populations emerged as significantly associated with cognitive function. Within the B cell compartment, *IgD^−^ CD27^−^ %lymphocyte* appeared to be potentially neuroprotective, whereas *CD27^+^ memory B cells* and *CD38^+^ transitional B cells* were identified as possible risk factors for cognitive decline. Notably, our finding for *CD38^+^ transitional B cells* is consistent with a previous report where they were described as a protective factor against vascular dementia (Chang et al. 2024), suggesting a similarly beneficial role for this cell type. Furthermore, within T cell maturation stages, *CCR7^+^ naive CD4^+^ T cells* demonstrated beneficial associations with cognitive performance, whereas HLA‐DR expression on *CD14^−^CD16^−^ monocytes* correlated negatively with cognitive outcomes.

Human peripheral B lymphocytes comprise four major subsets based on CD27 and IgD expression: *unswitched memory B cells (CD27^+^IgD^+^)*, associated with early immune responses; *switched memory B cells (CD27^+^IgD^−^)*; *naive/transitional B cells (CD27^−^IgD^+^)* from recent bone marrow generation; and *double negative B cells (CD27^−^IgD^−^)*, a heterogeneous population linked to aging and systemic lupus erythematosus. These distinct B cell compartments exhibit specialized functions in health and disease states (Castleman et al. 2023; Sanz et al. 2019).

Our data reveal a notable contrast wherein *unswitched memory B cells (CD27^+^ IgD^+^)* and *IgD^−^ CD27^−^ B cells* exhibit opposite relationships with cognitive metrics. This pattern warrants exploration in the context of current knowledge. Research indicates that physical activity in elderly individuals affects circulating immune profiles, with inactive seniors showing higher unswitched memory B cell frequencies than active counterparts. Given that these cells are considered indicators of immunological vitality (Poinsatte et al. 2019), their negative association with cognitive function in our study presents an unexpected paradox. Our observations regarding *IgD^−^ CD27^−^ B cells* also diverge from existing evidence. Previous investigations have documented elevated *Double Negative (IgG^+^IgD^−^CD27^−^) B cells* in Alzheimer's disease patients, exhibiting inflammatory receptor characteristics (Bulati et al. 2015; Martorana et al. 2014). Additionally, research has shown that *CD27^−^ IgD^−^ B cell* populations exert inflammatory effects in elderly Chinese males, correlating with functional deterioration (Nevalainen et al. 2019). Despite these apparent inconsistencies with published findings, the exact mechanisms by which these B cell subsets influence cognition remain undetermined, highlighting the necessity for mechanistic studies examining these immune‐cognitive pathways.

Our analysis revealed that *IgD^−^ CD27^−^ B cells* exhibit potential cognitive‐protective characteristics, with patterns similar to those observed in *IgD^−^ CD27^−^ lymphocyte* populations. Conversely, *CD27 expression on memory B cells* and *CD38 on transitional B cells* demonstrated associations with cognitive decline. Interestingly, previous investigations have documented significantly reduced *CD27^+^ B cell* percentages in elderly subjects (mean: 19.2%) compared to younger individuals (mean: 28.2%) (Chong et al. 2005). Furthermore, cells with high CD38 expression appear to confer protective effects against aging‐related processes (Biragyn et al. 2017). Within T cell maturation stages, CCR7 expression on naive *CD4^+^ T cells* correlates positively with cognitive function. The diminished vitality of naive *CD4^+^ T cells* observed in elderly individuals with cognitive deterioration (Moro‐García et al. 2013) suggests these cells may function as potential cognitive‐protective factors. Regarding monocytes, *HLA‐DR expression on CD14^−^CD16^−^ cells* demonstrates negative associations with cognitive performance. Notably, research indicates HLA‐DR expression on monocytes increases with advancing age, suggesting HLA‐DR‐expressing monocytes might represent potential cellular targets for cognitive enhancement interventions (Busse et al. 2015).

Our bidirectional MR analyses detected no significant reverse causal effects from cognitive performance to the identified seven potential immune phenotypes. This absence of reverse causality reinforces our conclusion that these immune signatures influence cognitive function rather than the opposite. This directional clarity enhances the potential value of these immune pathways as therapeutic targets for cognitive enhancement.

Several considerations should be noted when interpreting our findings. The reliance on GWAS data primarily from individuals of European descent inherently limits the immediate applicability of our conclusions to more diverse global populations. Methodologically, a central challenge within our MR framework is the potential for confounding from IV heterogeneity or pleiotropy, which could compromise the stability of our causal estimates. To address this proactively, we deployed a rigorous validation pipeline, including Cochran's *Q* tests for heterogeneity, leave‐one‐out analyses to detect influential SNPs, and visual funnel plot inspections for asymmetry (Table S5, Figures S1–S13). In cases with an adequate number of instruments, formal assessments via the MR‐Egger intercept and MR‐PRESSO did not reveal evidence of directional pleiotropy (Tables S6 and S7). A crucial caveat, however, is that the applicability of these latter tests is contingent on the number of SNPs; consequently, findings derived from a sparse set of instruments carry a higher burden of potential pleiotropic confounding and thus warrant a more cautious interpretation. Beyond these statistical considerations, our MR approach, although suggestive of causality, cannot elucidate the underlying molecular pathways, which await experimental validation.

In conclusion, our MR analysis provides evidence suggesting potential causal effects of specific immune cell profiles on cognitive function. These findings point to potential immune‐related pathways and biomarker candidates for cognitive assessment.

## 5 Conclusion

Our two‐sample MR investigation has suggested potential causal relationships between immune cell traits and cognitive abilities, elucidating the directional influence from the immune system to cognitive processes. The bidirectional analyses we conducted verified no reverse causality, reinforcing our findings. This work advances our comprehension of the mechanisms underlying cognitive performance and highlights potential immune pathways that could inform future intervention research.

## Author Contributions

**Jingfeng Fu**: conceptualization, data curation, formal analysis, funding acquisition, software, supervision, writing – original draft, writing – review and editing. **Minmin Yang**: writing – review and editing. **Qingteng Zheng**: writing – review and editing.

## Conflicts of Interest

The authors declare no conflicts of interest.

## Peer Review

The peer review history for this article is available at https://publons.com/publon/10.1002/brb3.70861.

## Supporting information




**Supplementary Tables**: brb370861‐sup‐0001‐TableS1‐S8.xlsx

**Supplementary Figures**: brb370861‐sup‐0002‐FiguresS1‐S13.docx

## Data Availability

The datasets analyzed in this study include publicly available summary statistics. GWAS summary data for immune cell phenotypes are accessible from the IEU Open GWAS Project (accession numbers ebi‐a‐GCST90001391 to ebi‐a‐GCST90002121). GWAS summary statistics for cognitive performance can be accessed through the IEU Open GWAS Project (https://gwas.mrcieu.ac.uk/datasets/ebi‐a‐GCST006572/). All results from our analyses are presented in the main text and Supporting Information section. Questions about data access can be directed to the corresponding authors. Data access is subject only to restrictions imposed by the original data governing committees.
