# Supplementary material for: Causal Effects of Immune Cell Populations on Cognitive Performance: A Mendelian Randomization Study
Source: Brain Behav. 2025 Sep 10;15(9):e70861. doi: 10.1002/brb3.70861 (PMC12423432; doi:10.1002/brb3.70861)
Supplement: Supplementary file 2 — Supplementary Figures: brb370861‐sup‐0002‐FiguresS1‐S13.docx [file BRB3-15-e70861-s001.docx]

**Supplementary Figures**


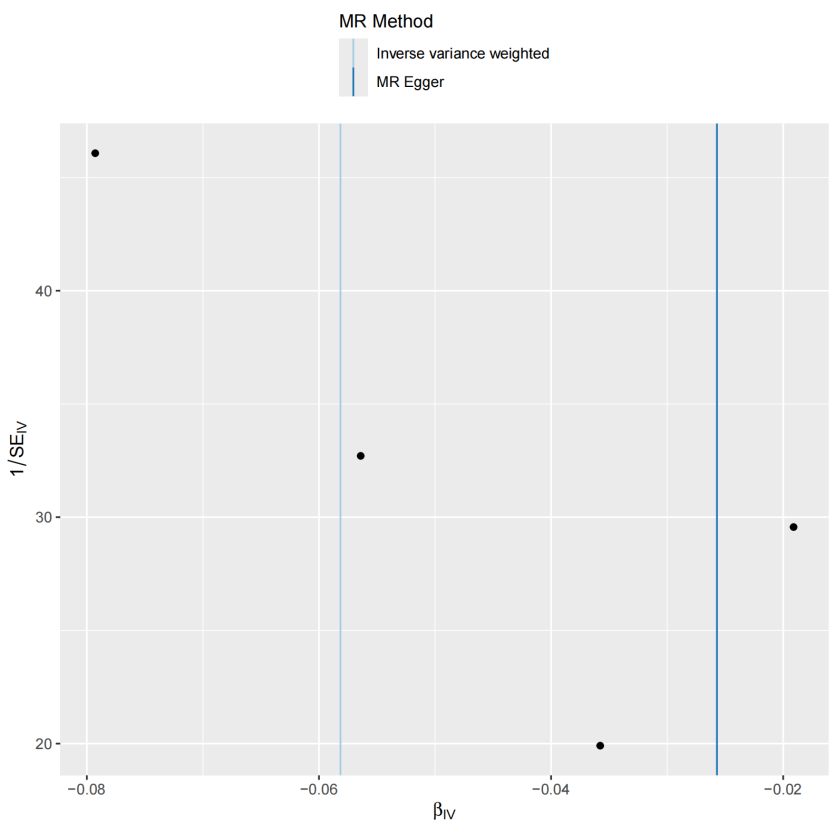


**Figure S1.** Funnel plots for *Unsw Mem %lymphocyte.*


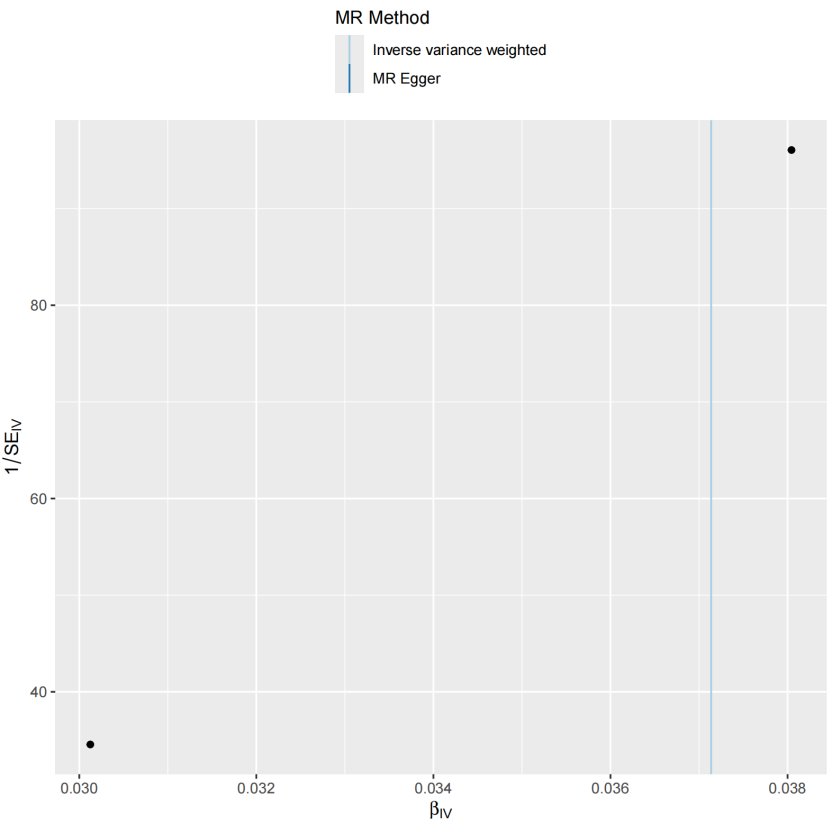


**Figure S2.** Funnel plots for *IgD- CD27- %lymphocyte.*


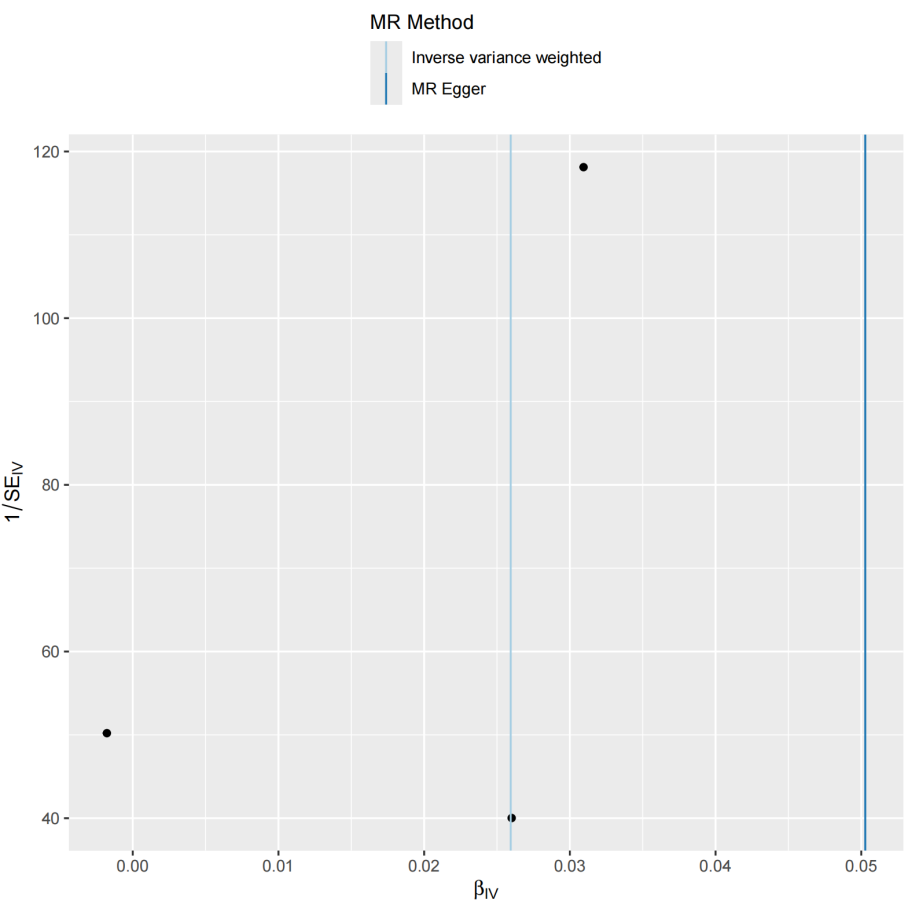


**Figure S3.** Funnel plots for *IgD- CD27- %B cell.*


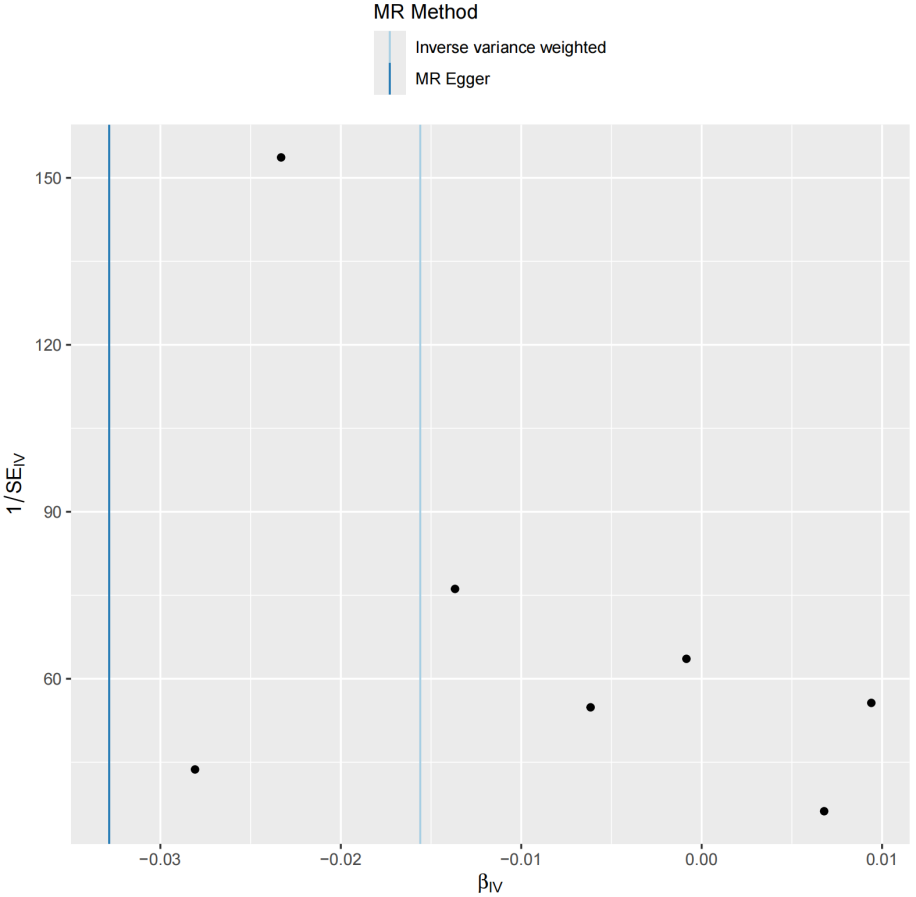


**Figure S4.** Funnel plots for *CD27 on memory B cell.*


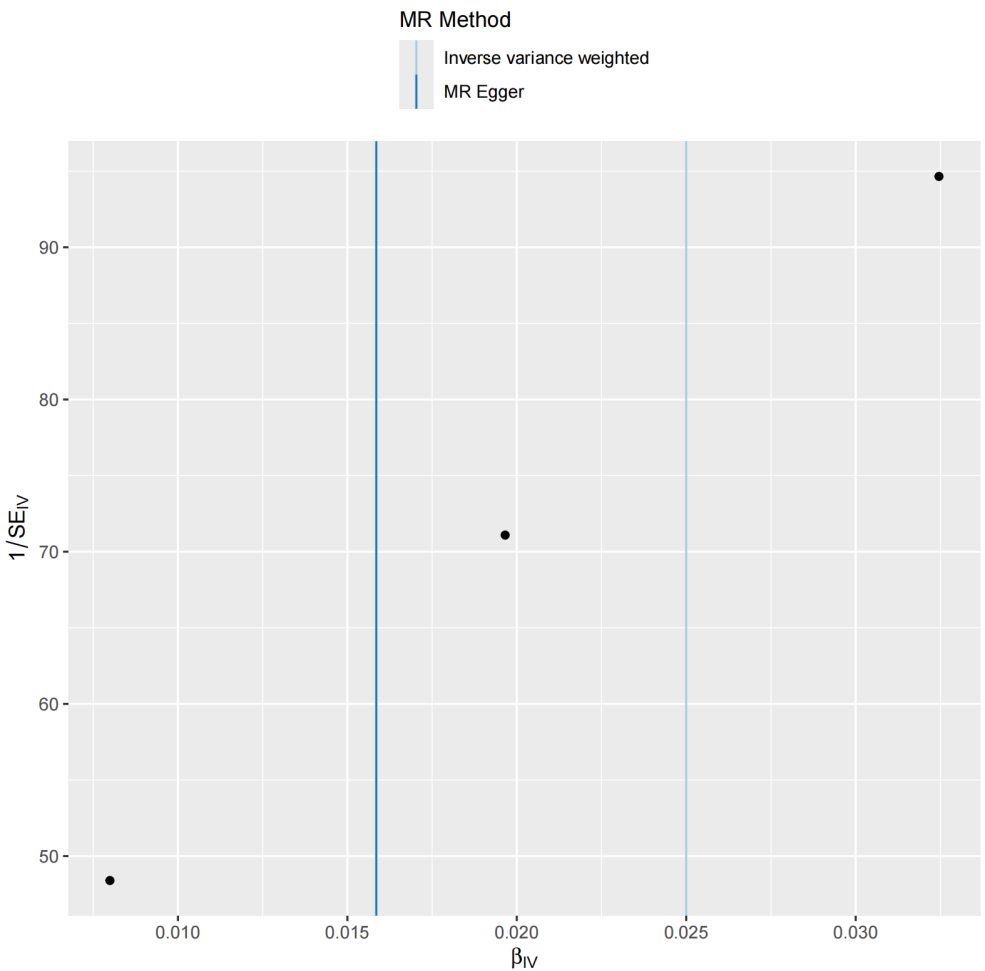


**Figure S5.** Funnel plots for *CCR7 on naive CD4+.*


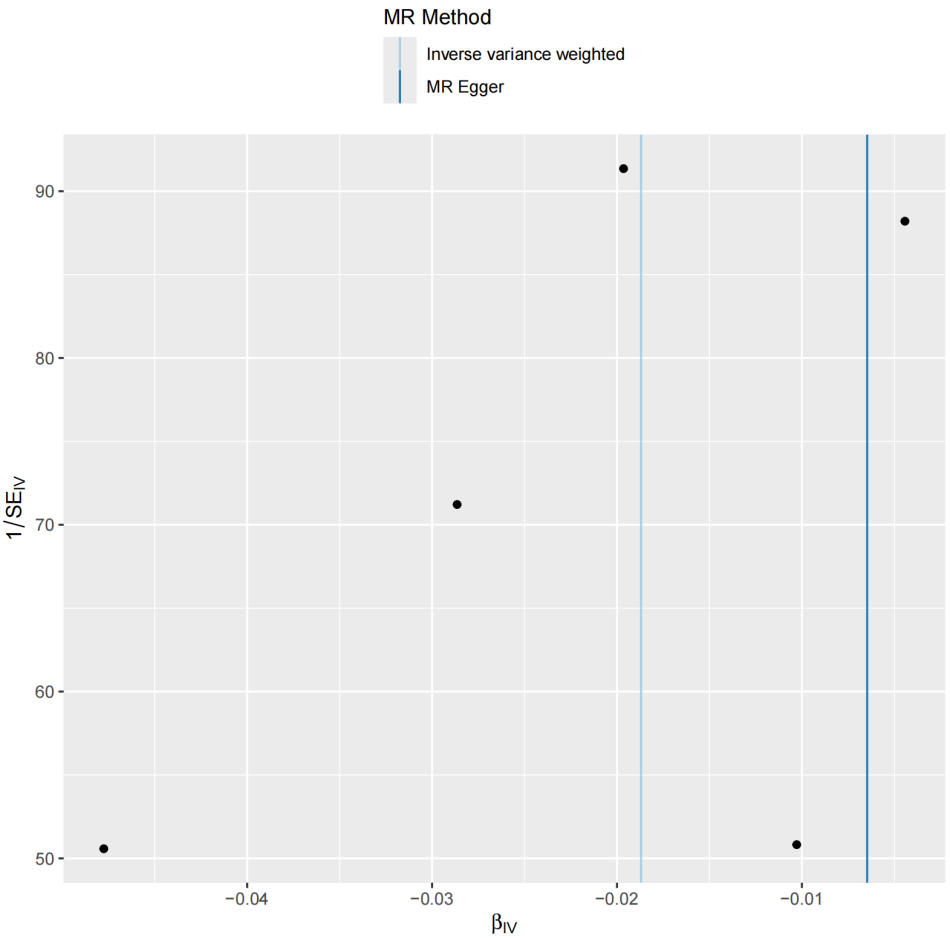


**Figure S6.** Funnel plots for *CD38 on transitional*.


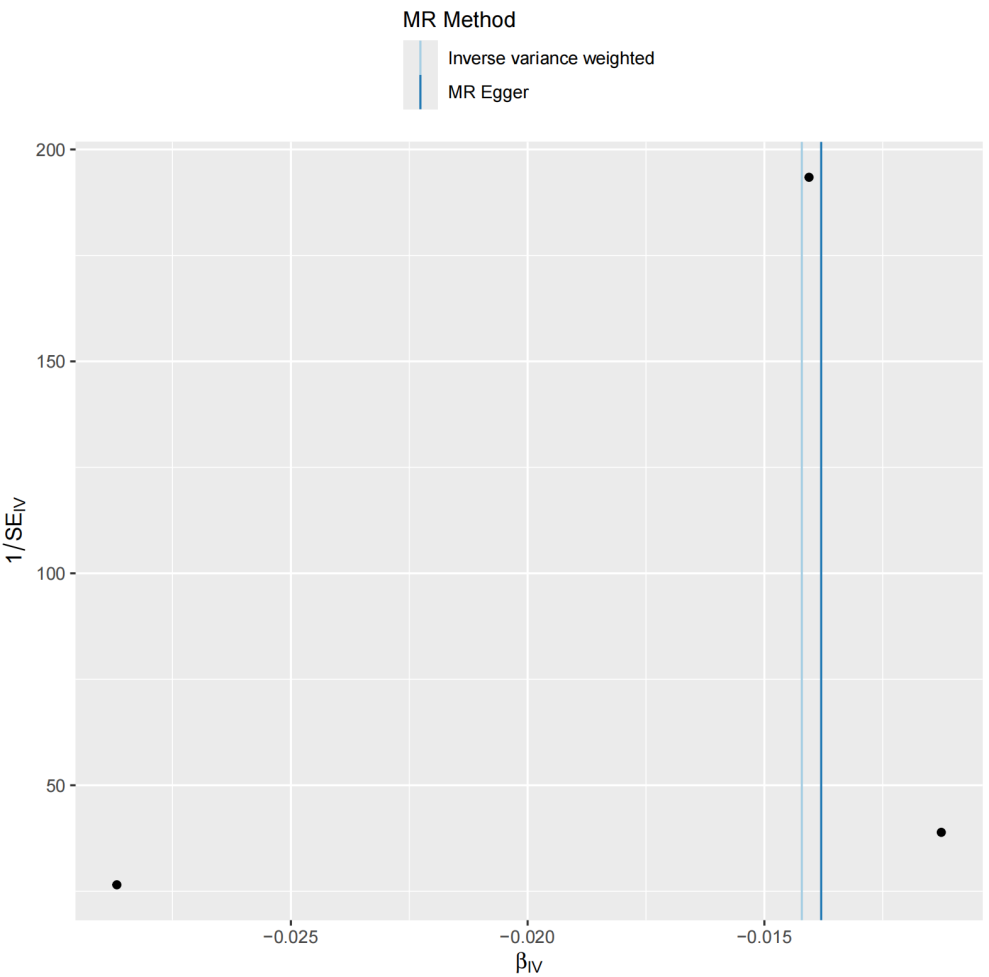


**Figure S7.** Funnel plots for *HLA DR on CD14- CD16-.*


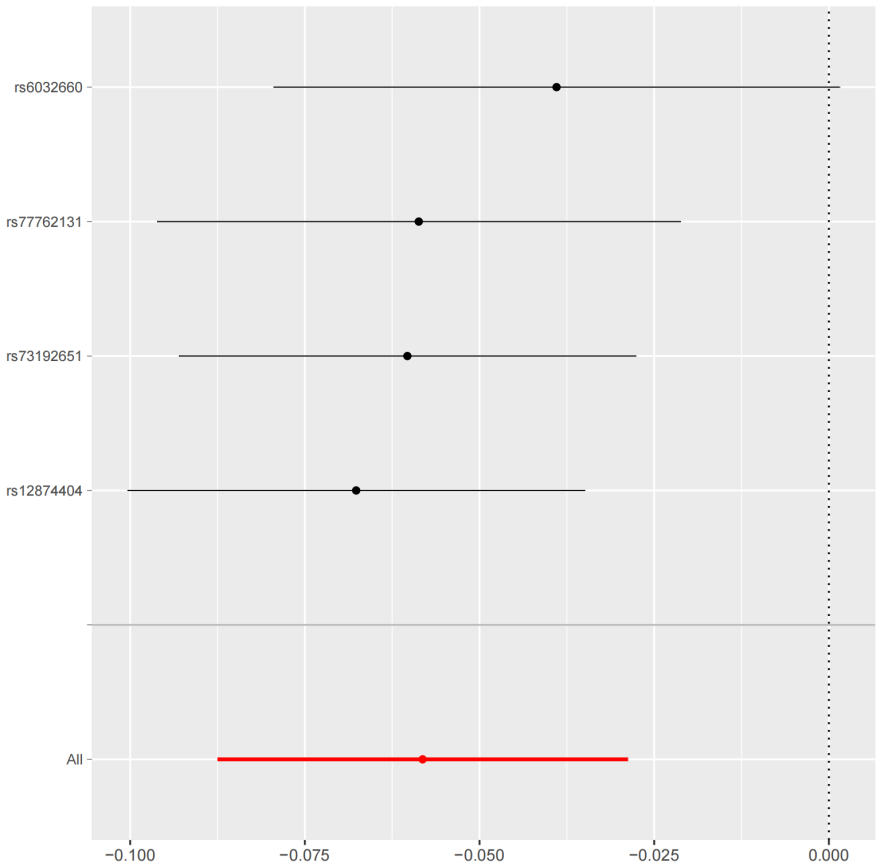


**Figure S8.** leave-one-out sensitivity analyses for *Unsw Mem %lymphocyte.*


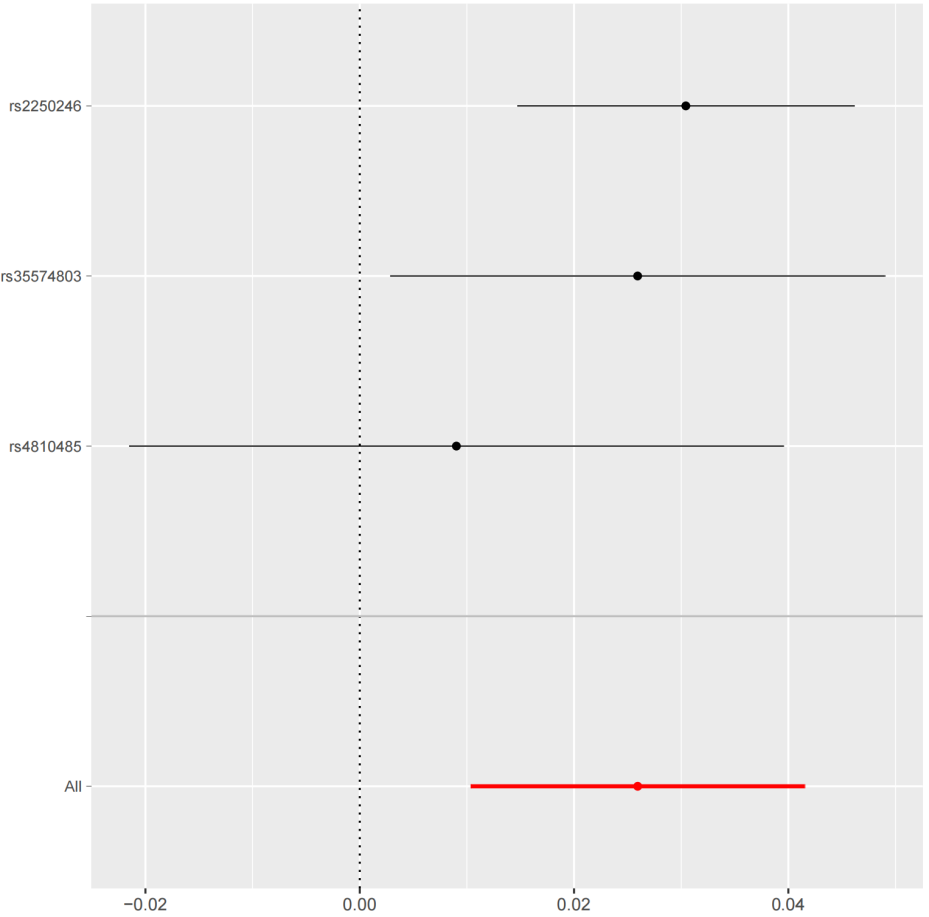


**Figure S9.** leave-one-out sensitivity analyses for *IgD- CD27- %B cell.*


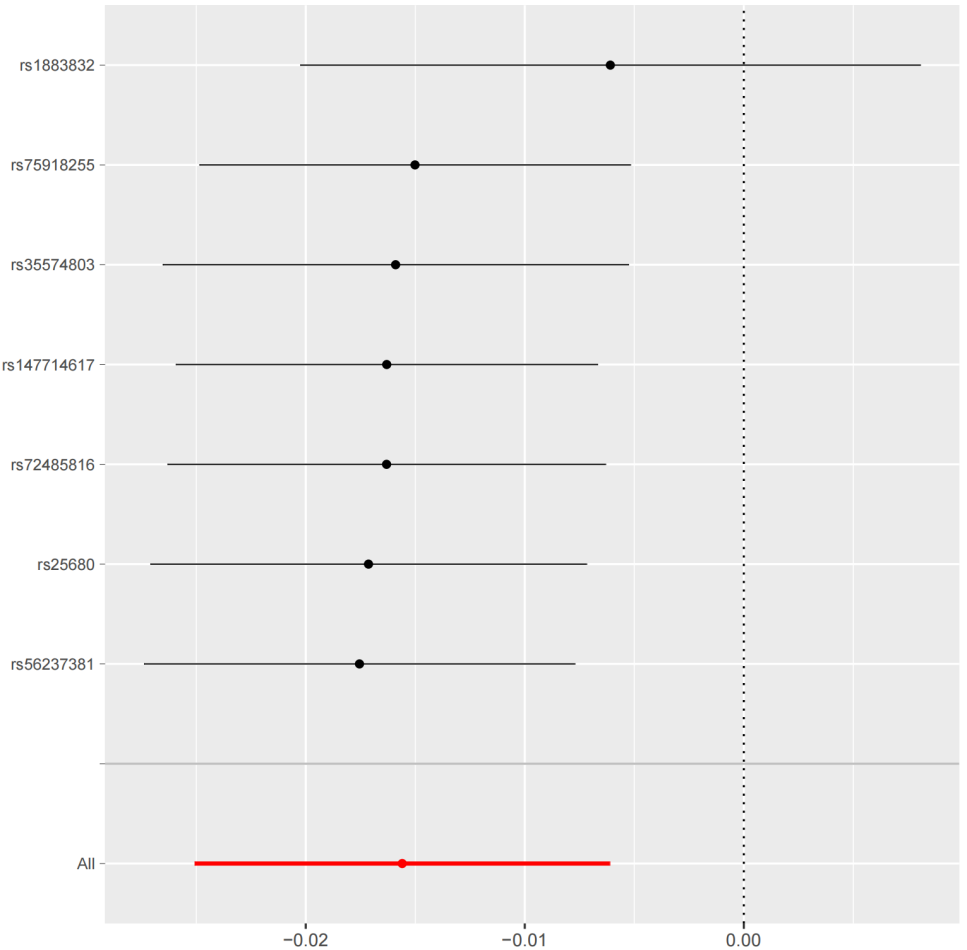


**Figure S10.** leave-one-out sensitivity analyses for *CD27 on memory B cell.*


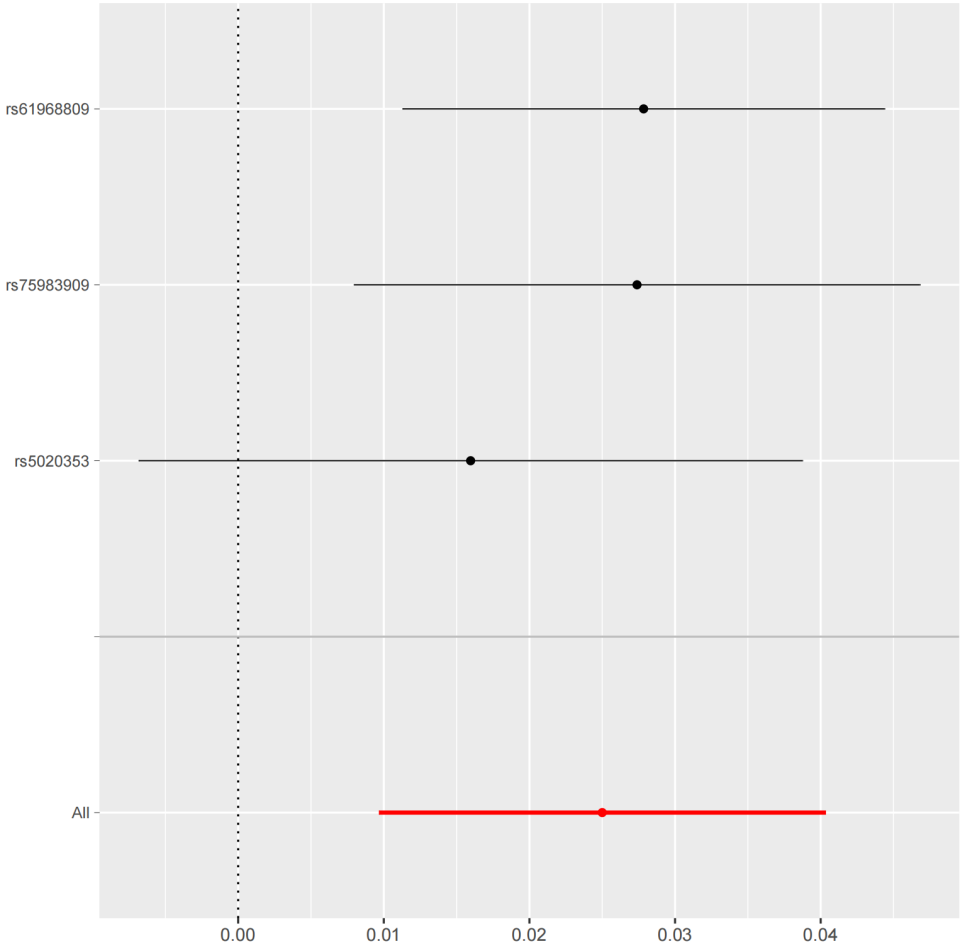


**Figure S11.** leave-one-out sensitivity analyses for *CCR7 on naive CD4+.*


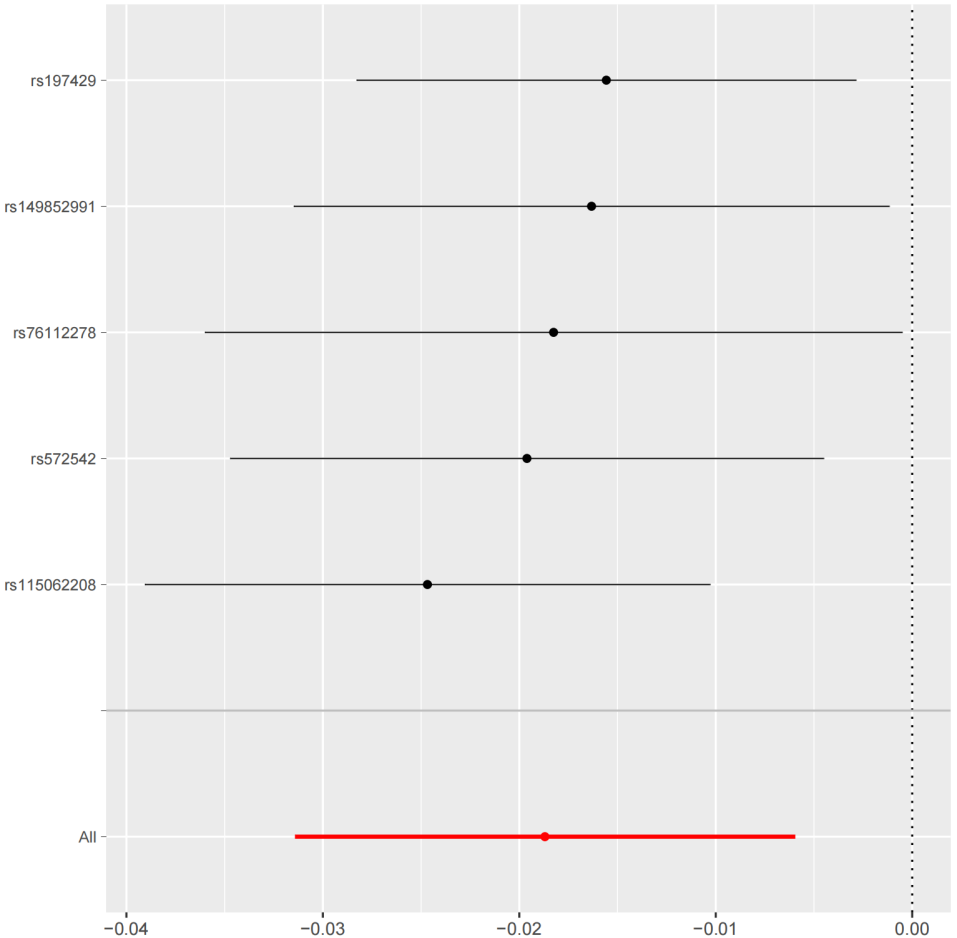


**Figure S12.** leave-one-out sensitivity analyses for *CD38 on transitional.*


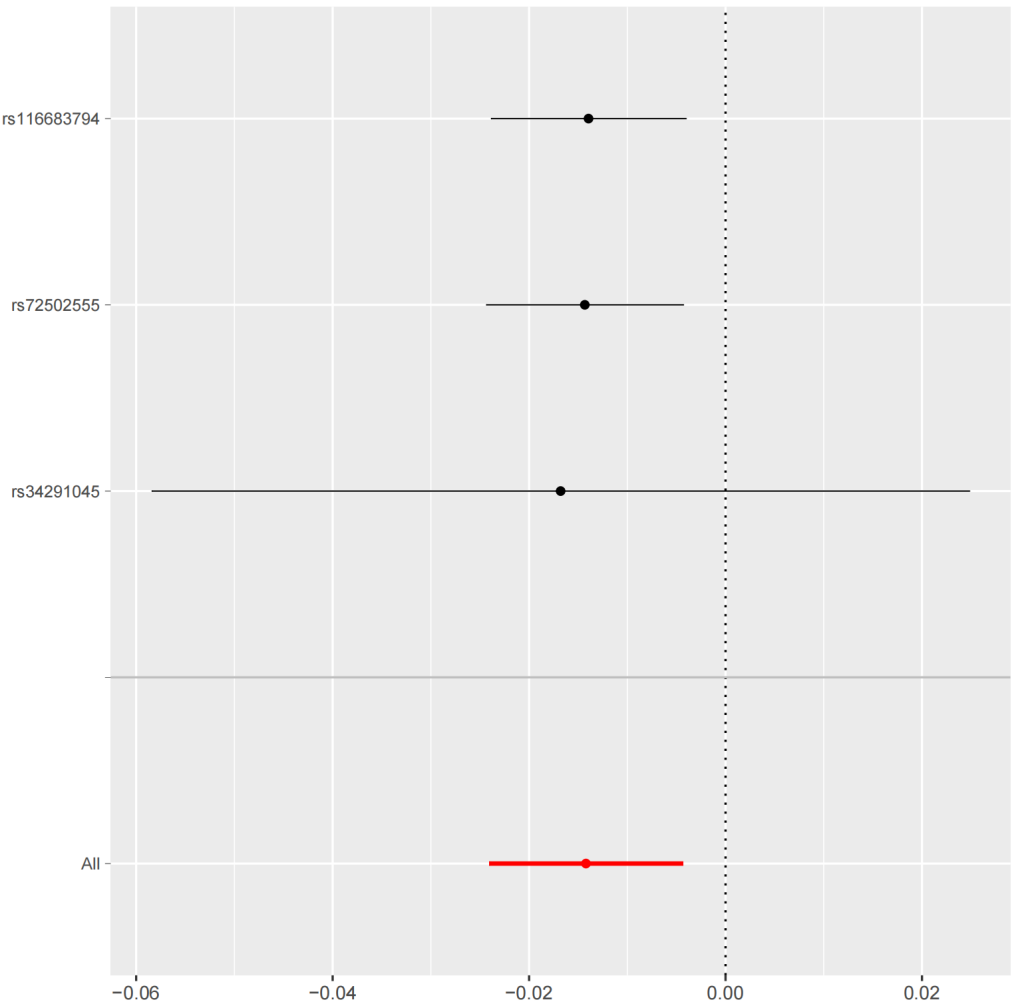


**Figure S13.** leave-one-out sensitivity analyses for *HLA DR on CD14- CD16- .*
